# Analyzing Mortality Patterns and Location of Death in Patients With Malignant Esophageal Neoplasms: A Two-Decade Study in the United States

**DOI:** 10.7759/cureus.50455

**Published:** 2023-12-13

**Authors:** Sreejith Vijayakumar, Abirami Saravanan, Nailah Sayeed, Nicole Gabriella Rusizana Kirezi, Nirupam K Duggirala, Ahmed H El-Hashash, Hussein Al Hussein

**Affiliations:** 1 Internal Medicine, Government T.D. Medical College, Alappuzha, IND; 2 Internal Medicine, Sree Gokulam Medical Center, Attingal, Thiruvananthapuram, IND; 3 Surgery, Texila American University, Georgetown, GUY; 4 Internal Medicine, Deccan College of Medical Sciences, Hyderabad, IND; 5 Internal Medicine, Wenzhou Medical University, Wenzhou, CHN; 6 Internal Medicine, Sree Balaji Medical College and Hospital, Chennai, IND; 7 Medicine, Charles University, Prague, CZE; 8 Internal Medicine, Hamad Medical Corporation, Doha, QAT

**Keywords:** racial disparities, gender, age, location, mortality patterns, esophageal neoplasms

## Abstract

Background

Esophageal neoplasm carries significant implications for end-of-life care. Despite medical advancements, disparities in the location of death persist. Understanding the factors influencing the place of death for esophageal neoplasm patients is crucial for delivering patient-centered care.

Objectives

The primary objective of this study is to inspect and evaluate mortality patterns in patients with malignant esophageal neoplasms over the past two decades.

Materials and methods

Using the CDC-WONDER database, the authors analyzed 309,919 esophageal neoplasm-related deaths. Data was categorized by age, gender, race, and location of death, enabling a detailed examination of the factors influencing the place of death.

Result

This analysis revealed significant disparities in death locations. Age, gender, race, and geographic region all played substantial roles in determining where esophageal neoplasm patients spent their final moments. Notably, males consistently experienced higher mortality rates across all settings. Geographic disparities indicated varying mortality rates by census region, with the Southern region reporting the highest rates. Racial disparities were also evident, with white individuals having the highest number of deaths.

Conclusion

This study underscores the importance of recognizing and addressing disparities in the place of death among esophageal neoplasm patients in the United States. By shedding light on the demographic influences on end-of-life decisions, it paves the way for more targeted and patient-centered approaches to end-of-life care for this patient population.

## Introduction

With an overall 5-year survival rate of less than 25%, cancer of the esophagus is the sixth most prevalent cause of death worldwide and the eighth most frequent diagnosis [1]. It happens more frequently in middle-aged and older males [1]. Worldwide aging and population increase, as well as an increasing number of risk factors like tobacco and alcohol use, a poor diet, inactivity, and obesity, are all contributing to a significant rise in the incidence and death of esophageal cancer [1]. Esophageal cancer can be deadly, with significant mortality rates and a dismal outlook at the point of diagnosis. Esophageal cancer is predicted to be diagnosed in 17,650 cases per year in the USA, with 16,080 fatalities anticipated [2].

Most people with malignant neoplasms favor a gentler approach to care in the final stages of their lives [3]. Although patients and their families want to relieve the patients' suffering and prevent them from being kept functioning by machines and devices in hospitals, many patients pass away in high-intensity care settings with invasive procedures and extensive testing. This places a greater emphasis on quantity than quality of life, which may delay the timely transfer to personalized care and lengthen hospital stays [3]. It is crucial to remember that vigorous treatment does not always increase survival and is frequently associated with a decline in patients' quality of life and a greater emotional toll on their families. Only 40% of patients in the US pass away at home or in a hospice, despite the fact that about 85% of them are inclined to [4].

Evaluation and identification of disparities in places of death is an indispensable guide for physician and patient education as place of death is often used as an indicator for end-of-life care quality [3]. This will also increase the probability of cancer patients receiving end-of-life care that aligns with their core values and can help physicians achieve patient-centered goals.

The primary objective of this study is to inspect and evaluate mortality patterns in patients with malignant esophageal neoplasms over the past two decades. Taking age, gender, racial background, and census regions in the United States of America into consideration, discrepancies in the location of death, such as medical and nursing facilities, home and hospice care, were assessed.

## Materials and methods

This cross-sectional study examined differences in malignant esophageal neoplasm mortality throughout the United States of America. The Centre for Disease Control and Prevention's Wide-ranging Online Data for Epidemiologic Research, or CDC-WONDER, provided the information for this analysis. WONDER is an online platform that provides the public and public health professionals with access to the resources of the Centre for Disease Control and Prevention (CDC). Access to a vast diversity of public health information is made possible by the system. [5]

The data obtained was from 1999 to 2020, under the section of the underlying cause of Death by Bridged-Race categories, and was extracted on August 27th, 2023. The ICD-11 (International Classification of Disease, Eleventh Revision) code number selected was C15 (Malignant neoplasm of esophagus).

The CDC-WONDER database has several sub-categories in the place of death. The deaths in Medical facility-inpatient, Medical facility - Outpatient or Emergence Room, Medical Facility - Dead on arrival, Medical facility - Status unknown, and Nursing were combined as "Hospital"; those as descendant's home as "Home", those in Hospice facility as "Hospice" and Other. 

The authors used four variables in the assessment, which are, all ages (ten-year age groups), genders, four census regions of the USA (Northeast, Midwest, South, and West), and all races.

The frequency polygons of home or hospice deaths trends were obtained by CDC-WONDER. Yearly mortality rate was charted for the overall death trends (from 1999-2020) and for forecasting (from 1999-2025) adding five years of prediction. The method used is called Autoregressive Integrated Moving Average (ARIMA) model. Yearly death rates were also charted for all age groups, all genders, in four census regions, and all races.

## Results

This analysis of 309,919 esophageal neoplasm-related deaths between 1999 and 2020 obtained from the CDC-WONDER database revealed several significant findings, which are discussed below. 

Table 1 shows data on the place of death categorized by ten-year age groups, gender, US census region, and race. The maximum number of deaths occurring in home or hospice settings was observed in the 65-74 years age group (n=46,243), whereas the minimum number of deaths was observed within the 15-24 years age group (n=46). Similar patterns were evident in the category of medical facilities and nursing homes as the place of death. The maximum number of deaths in this category occurred among individuals in the 65-74 years age group (n=41,677), whereas the minimum number of deaths was seen in the 15-24 years age group (n=34). In the last category - Others - the maximum number of deaths was again seen in the 65-74 years age group (n=4075), whereas the 15-24 years age group had zero deaths. When analyzing the data by gender, it is evident that males consistently experienced a higher number of deaths compared to females in all three settings: Home or hospice (n=122788), Medical facility or nursing home (n=110145), and Others (n=11551). Examining the data based on the US census region, it becomes apparent that the maximum number of deaths across all three settings was observed in US census region 3, which corresponds to the South. The Northeast region had the lowest number of deaths in both the Home or hospice (n=27770) and Others (n=1899) settings. In the medical facility and nursing home settings, the West had the minimum number of deaths (n=24918). Across all three categories of the place of death, the data reveals that individuals identified as white had the highest number of deaths. Conversely, American Indian/Alaskan Native individuals had the lowest number of deaths.

**Table 1:** The data on the place of death categorized by ten-year age groups, gender, US census region, and race.

	Home or Hospice (n = 153696)	Medical Facility or Nursing (n = 140652)	Others (n = 15571)
Ten-Year Age Groups			
15-24 years	46	34	0
25-34 years	365	350	39
35-44 years	2637	2601	299
45-54 years	14194	13702	1603
55-64 years	37213	33545	3480
65-74 years	46243	41677	4075
75-84 years	37231	34223	4031
85+ years	15753	14500	2039
Gender			
Female	30908	30507	4020
Male	122788	110145	11551
Census Region			
Census Region 1: Northeast	27770	33413	1899
Census Region 2: Midwest	37824	36193	3736
Census Region 3: South	55699	46119	7086
Census Region 4: West	32403	24918	2850
Race			
American Indian or Alaska Native	659	739	60
Asian or Pacific Islander	2194	2651	229
Black or African American	11523	17932	1684
White	139320	119325	13586

Table 2 shows home or hospice death predictors in the case of esophageal cancer. Univariate logistic regression analysis of collected data reveals that individuals aged 65-74, males, patients residing in Census Region 4 (West), and of white race were significantly more likely to experience home or hospice deaths. The 65-74 years age group was found to be 1.061 times more likely to have deaths compared to the 85+ years age group (reference). Males exhibit a 1.127-fold higher likelihood of experiencing home or hospice death compared to females. In contrast to Census Region 1, representing the Northeast, Region 4 - West exhibits a significantly 1.484-fold higher likelihood of having home or hospice deaths.

**Table 2:** Home or hospice death predictors in the case of esophageal cancer. The significant P-values (<0.05) are marked with an asterisk (*).

Variables	Univariate Logistic Regression		
	Odds Ratio	95% Confidence Interval	P-value
Age			
15-24 years	1.42	(0.911, 2.214)	0.121
25-34 years	0.985	(0.853, 1.138)	0.839
35-44 years	0.955	(0.902, 1.011)	0.111
45-54 years	0.974	(0.943, 1.005)	0.098
55-64 years	1.055	(1.028, 1.083)	<0.001*
65-74 years	1.061	(1.035, 1.088)	<0.001*
75-84 years	1.022	(0.996, 1.049)	0.105
85+ years	1.0 (Reference)		
Gender			
Male	1.127	(1.108, 1.147)	<0.001*
Female	1.0 (Reference)		
Census Region			
Census Region 1: Northeast	1.000 (Reference)		
Census Region 2: Midwest	1.205	(1.179, 1.23)	<0.001*
Census Region 3: South	1.331	(1.305, 1.358)	<0.001*
Census Region 4: West	1.484	(1.451, 1.518)	<0.001*
Race			
American Indian or Alaska Native	1.404	(1.263, 1.561)	<0.001*
Asian or Pacific Islander	1.297	(1.221, 1.377)	<0.001*
White	1.784	(1.742, 1.828)	<0.001*
Black or African American	1.000 (Reference)		

**Figure 1:**
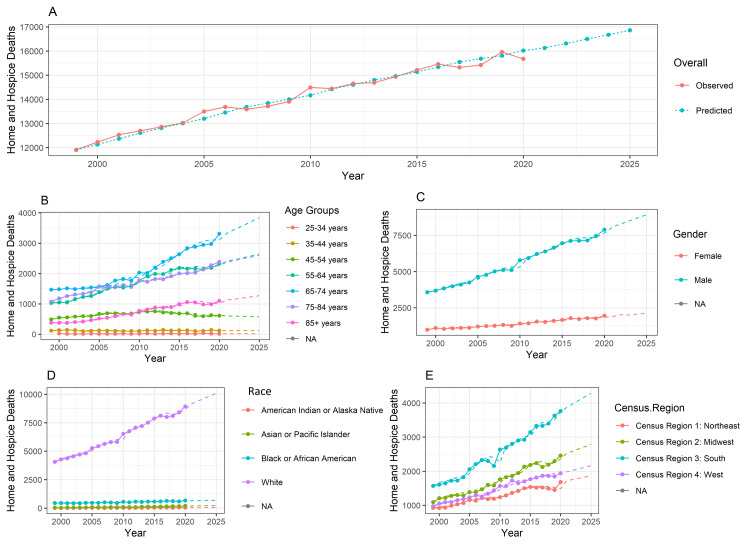
Home or hospice death trends in esophageal cancer-related deaths from the year 1999 to 2020. The forecasting is done from year 1999 to 2025. The training data is available from year 1999 to 2020. So, the prediction is done for another 5 years. In the line chart, the lines represent the observed data. The dotted line represents the forecasted data. The method used is the Autoregressive Integrated Moving Average (ARIMA) model.

Figure 1 shows home or Hospice death trends in esophageal cancer-related deaths from the year 1999 to 2020. In Figure 1A, there is a steady increase in such deaths over time, with occasional fluctuations. Additionally, the predictive trend calculated using the ARIMA model suggests that such deaths are expected to continue increasing in the coming years, potentially until 2025. Figure 1B highlights a growing number of home or hospital deaths in the 65-74 years age group. Figure 1C depicts an increasing mortality trend in male gender compared to females. Figure 1D reveals that the white racial group had the highest number of deaths compared to other races. Figure 1E shows US census region 3 - South had the highest number of home or hospice deaths.

## Discussion

Cancer is a daunting diagnosis for most people. Improving end-of-life care for cancer patients has been an increasing research and teaching goal over the past two decades. [6,7]. It is critical for healthcare professionals to understand the perceptions of life and death of end-of-life cancer patients since they form the foundation of treatment and influence the place of death in such patients. The ability to die in a desired setting is a vital component of high-quality cancer care. Preference for location of care and death is not a fixed idea and can vary over time because of discussions between healthcare experts and patients. [8,9]

A 22-year data set was gathered from CDC WONDER to study the mortality trends of esophageal neoplasms. The authors identified trends and discrepancies in place of death in this study of 309,919 patients who died from esophageal cancer between 1999 and 2020. Prior evidence by Bajaj et al has confirmed that significant disparities exist in the location of death based on age, race, and sex, and this study adds to this by confirming these findings and also including census regions as a parameter, which was not done by the previous study [10].

According to these findings, almost 50% of patients with esophageal neoplasm died at home or in hospice, compared to 45% who died in a medical or nursing facility and 5% who died elsewhere. It has been demonstrated in the past, in line with these findings, that half of cancer patients in their later years prefer to pass away at home [11].

Higginson and Sen-Gupta recognized that individuals with advanced cancer do not like to die in institutionalized settings, with death at home being the most prevalent desire followed by hospice [12,13]. Despite these preferences and the superior results of care at home compared to other settings, only a few died at home. The reason can be that patients may be transferred to subacute or acute care settings before passing away even though they choose to die at home because of a lack of caregiver support, a lack of healthcare provider knowledge of preferences, and poor symptom control. [14,15] Although there are many factors that determine where people desire to die, we took into consideration those criteria that are more likely to have an impact.

These findings add to the emerging evidence from recent observational studies [16] that the highest death rates occur in older age groups (65-74 years old) in every setting, be it home, hospice, medical or nursing facility, or elsewhere. This is most likely due to the increased prevalence of esophageal neoplasm in older age groups even though there is promising evidence of a decreasing trend in incidence in this age group [17].

As per our study, the chances of home or hospice death were highest in the age group 15-24 years old. Despite being rare, young-onset esophageal cancer is becoming more common [18]. It is alarming that the percentage of advanced disease is rising. Young-onset esophageal adenocarcinoma also manifests at later stages, leading to a worse prognosis for remaining cancer-free [18]. Gender further influences the age-adjusted mortality rates. Males were four times more likely to die due to the condition than females in this study. The extraordinarily high sex ratios may be partially attributed to specific risk factors, such as smoking and alcohol intake in males [19] and the supposedly protective effect of estrogen in females [20-23].

The authors discovered significant geographic variation in mortality rates by census region in this analysis. The highest mortality rates were observed in the Southern region, which accounts for one-third of total deaths, and the lowest mortality rates were observed in the West, which constituted around one-fifth of the total deaths. The regional variance in cancer burden was most likely caused by differences in obesity rates [24], smoking, and alcohol usage [25]. It is also plausible that geographical heterogeneity in esophageal neoplasm is driven by differential exposure to a strong, widespread, and as-yet unexplained causative factor, as Kubo et al. proposed, which has resulted in a substantial increase in disease incidence [26].

However, an intriguing discovery was that the North East region had more fatalities in medical or nursing facilities than hospice, in contrast to the other regions where hospice deaths outnumbered other death locales. Previous studies on geographic disparities in esophageal cancer mortality relied on data with limited geographic spread and focused on rates in specific cancer registries [27].

Similarly, racial disparities in the mortality rates were also observed by us. We noted that more than four out of five deaths were white population, and more than half of the whites died in home or hospice. The other races, including Black or African American, Asian or Pacific Islander, and American Indian or Alaska Native (AIAN) had more deaths in a medical or nursing facility than home or hospice. Racial/ethnic health disparities are multifaceted, including socioeconomic hurdles, a history of discrimination in health care, and cultural differences. Ethnic minorities have the greatest poverty rates, which typically results in fewer options for hospice or nursing home selection [28]. Minority communities have inequitable access to these care options, which may contribute to higher utilization of acute end-of-life services and thus more deaths in hospitals or nursing homes [29]. Whether these disparities are primarily attributable to disparities in the availability of palliative care services or to variations in care preferences, with Blacks favoring hospital death and life-prolonging therapies over whites, is unclear [30]. The fact that trends show a decrease in hospital mortality over time while also showing an increase in deaths at home and in hospice is encouraging. Similar to this study, Bajaj et al were concerned about the fact that ethnic minorities and persons of color had a lower likelihood of passing away at home or in palliative care than White decedents [10].

Overall, this study points out various disparities regarding the place of death in patients with esophageal neoplasm in the US, and these discrepancies are pervasive throughout the time period of 1999 to 2020. This study highlights the need for more investigation into the underlying psychological, social, and systemic causes of differences in where people die.

Limitations

No data sets from the most recent years, 2021-2023, are reported in this study. Another limitation is that CDC WONDER is an online database that relies on death certificates. Any inaccuracies in the coding of death certificates may distort the results.

## Conclusions

The number of deaths at home or hospice from esophageal cancer in the USA is increasing. This conclusion holds even when stratified for age, race, gender, and census region with a few exceptions (non-white populations and the 45-54 years old age group). These exceptions, however, must be studied further as they may point us in the direction of finding which future measures are necessary for oncologists to use to prolong life. Furthermore, further stratification by other potential confounders is required such as socioeconomic status, access to healthcare, and treatment type. Longitudinal studies are also necessary to verify conclusions derived from this study.
